# Plasminogen Controls Inflammation and Pathogenesis of Influenza Virus Infections via Fibrinolysis

**DOI:** 10.1371/journal.ppat.1003229

**Published:** 2013-03-21

**Authors:** Fatma Berri, Guus F. Rimmelzwaan, Michel Hanss, Emmanuel Albina, Marie-Laure Foucault-Grunenwald, Vuong B. Lê, Stella E. Vogelzang-van Trierum, Patrica Gil, Eric Camerer, Dominique Martinez, Bruno Lina, Roger Lijnen, Peter Carmeliet, Béatrice Riteau

**Affiliations:** 1 VirPath, EA4610 Virologie et Pathologie Humaine, Faculté de Médecine RTH Laennec, Université Claude Bernard Lyon 1, Lyon, France; 2 Department of Virology, Erasmus Medical Center, Rotterdam, The Netherlands; 3 Laboratoire d'Hématologie, CBPE, Hospices Civils de Lyon, Lyon, France; 4 CIRAD, UMR CMAEE, Montpellier, France INRA, UMR1309 CMAEE, Montpellier, France; 5 INSERM U970, Paris Cardiovascular Centre, Paris, France; 6 Université Paris-Descartes, Paris, France; 7 Center for Molecular and Vascular Biology, KU Leuven, Leuven, Belgium; 8 Laboratory of Angiogenesis & Neurovascular Link, Vesalius Research Center, VIB, Leuven, Belgium; 9 Laboratory of Angiogenesis & Neurovascular Link, Vesalius Research Center, KU Leuven, Leuven, Belgium; 10 INRA, Nouzilly, Indre-et-Loire, France; Johns Hopkins University - Bloomberg School of Public Health, United States of America

## Abstract

Detrimental inflammation of the lungs is a hallmark of severe influenza virus infections. Endothelial cells are the source of cytokine amplification, although mechanisms underlying this process are unknown. Here, using combined pharmacological and gene-deletion approaches, we show that plasminogen controls lung inflammation and pathogenesis of infections with influenza A/PR/8/34, highly pathogenic H5N1 and 2009 pandemic H1N1 viruses. Reduction of virus replication was not responsible for the observed effect. However, pharmacological depletion of fibrinogen, the main target of plasminogen reversed disease resistance of plasminogen-deficient mice or mice treated with an inhibitor of plasminogen-mediated fibrinolysis. Therefore, plasminogen contributes to the deleterious inflammation of the lungs and local fibrin clot formation may be implicated in host defense against influenza virus infections. Our studies suggest that the hemostatic system might be explored for novel treatments against influenza.

## Author Summary

Influenza viruses, including H5N1 bird influenza viruses continue to form a major threat for public health. Available antiviral drugs for the treatment of influenza are effective to a limited extent and the emergence of resistant viruses may further undermine their use. The symptoms associated with influenza are caused by replication of the virus in the respiratory tract and the host immune response. Here, we report that a molecule of the fibrinolytic system, plasminogen, contributes to inflammation caused by influenza. Inhibiting the action of plasminogen protected mice from severe influenza infections, including those caused by H5N1 and H1N1 pandemic 2009 viruses and may be a promising novel strategy to treat influenza.

## Introduction

Influenza A viruses (IAV) are an important cause of outbreaks of respiratory tract infections and are responsible for significant morbidity and mortality in the human population [1]. Upon infection with IAV, innate and adaptive immune responses are induced that restrict viral replication and that afford protection against infection with these viruses. However, excessive inflammation, particularly in the lower respiratory tract, may result in alveolar damage limiting respiratory capacity and deteriorate the clinical outcome of IAV infections [2], [3]. Dys-regulation of cytokine production in the lungs is thus often associated with a fatal outcome of IAV [4]. The sites of virus replication in the respiratory tract represent complex microenvironments, in which extracellular proteases are present abundantly [5], [6]. Some of these proteases can play a role in innate immune responses since they are important mediators of inflammatory processes [7] and influence virus replication [8], [9]. To date, however, the elucidation of host proteases contributing to pathogenesis of IAV infections *in vivo* has been hampered by the lack of experimental models.

One of the proteases of interest is plasmin, which is a serine protease involved in fibrinolysis, the biological process of dissolving fibrin polymers into soluble fragments. Plasmin is generated through cleavage of the proenzyme plasminogen, produced in the liver and present in the blood. Specific binding and conversion of plasminogen into plasmin by IAV may afford the virus an alternative protease for cleavage of its hemagglutinin molecule [10], [11]. This is an essential step in the virus replication cycle and this may contribute to the pathogenesis of IAV infection [12], [13]. In addition, plasminogen/plasmin plays a central role in fibrinolysis-mediated inflammation [14] and there is evidence of fibrinolysis activation during IAV infections [15]. Thus, plasminogen could contribute to the pathogenesis of IAV infections by promoting virus replication or by inducing a fibrinolysis-dependent harmful inflammatory response in the respiratory tract. At present it is unknown whether one or both of these two mechanisms of plasminogen activity contribute to pathogenesis of IAV infections *in vivo*. In the present study we address this research question and using plasminogen-deficient mice (PLG-KO) and pharmacological approaches the role of plasminogen during IAV infections was investigated.

Our findings show that plasminogen plays an important role in lung inflammation upon IAV infections, mainly through fibrinolysis. Therefore, targeting host factors, such as the fibrinolytic molecule plasminogen may be of interest for the development of new therapeutics against IAV infections.

## Results

### Plasminogen promotes IAV pathogenesis

**Figure 1:**
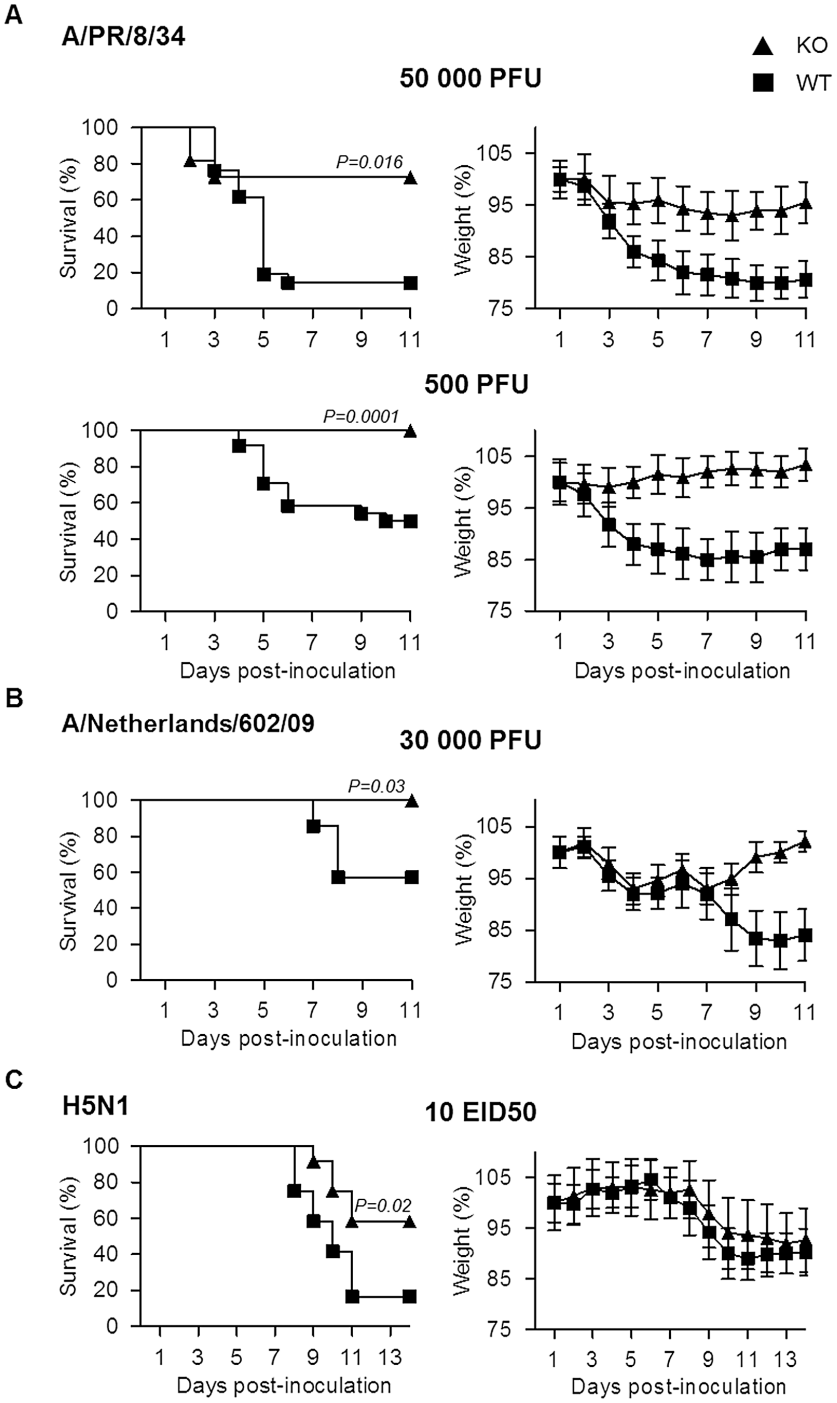
Plasminogen contributes to influenza pathogenesis. Survival and weight loss of PLG-KO (triangles) and WT (squares) mice infected with (A) IAV A/PR/8/34 (50,000 PFU; n = 11–21 or 500 PFU; n = 23–24), (B) A/Netherlands/602/09 (30,000 PFU; n = 7) or (C) A/chicken/Ivory-Coast/1787/2006 (10 EID50; n = 12). The proportion of survival was determined based on euthanasia criteria. Animals that lost 20% of their body weight were considered to have reached humane endpoints and were sacrificed according to the study protocol. It is of note that upon WT mice infection with A/chicken/Ivory-Coast/1787/2006, all infected mice lost weight but died before reaching −20% of their body weight, in contrast to PLG-KO mice, which explains the difference in mortality but not in weight loss. Weight loss data represent weight average ± s.e.m of the above indicated number of mice. n = mice per group.

To explore the role of plasminogen in IAV pathogenesis, we investigated the consequence of plasminogen-deficiency. Plasminogen +/− mice were intercrossed to generate wild-type (WT) and plasminogen −/− (PLG-KO) mice, which were infected with IAV A/PR/8/34 (H1N1; 50,000 or 500 PFU) and weight loss and survival rates were monitored. As shown in Figure 1A, compared to WT mice, PLG-KO mice were significantly more resistant to IAV-induced weight loss and death. In PLG-KO mice substantial protection was also observed against infection with 2009 pandemic virus A/Netherlands/602/09 (30,000 PFU, Figure 1B) and highly pathogenic H5N1 virus A/chicken/Ivory-Coast/1787/2006 (10 EID50 H5N1, Figure 1C). Of note, the latter was not adapted to replicate in mammals, which could explain the delay in weight loss observed upon infection, as also observed by others [16]. Thus, we concluded that without plasminogen, pathogenesis of IAV infections was dampened and mortality reduced in a subtype-independent manner.

### Protection conferred by PLG-deficiency is independent on virus replication

**Figure 2:**
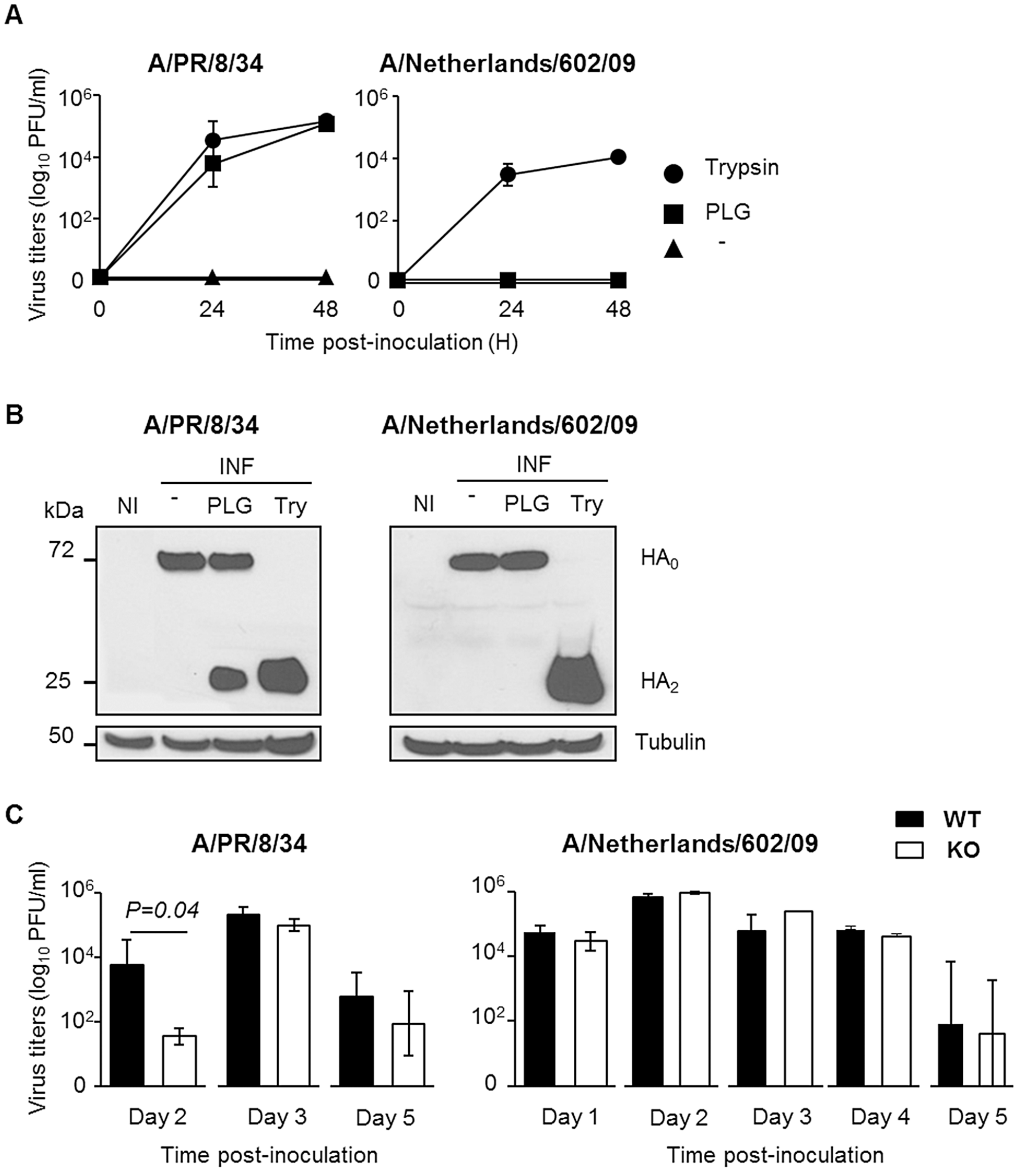
The deleterious role of plasminogen is independent on virus replication. (A) Virus replication of IAV A/PR/8/34 and A/Netherlands/602/09 after inoculation of A549 cells in presence or absence (triangle) of plasminogen (square) or trypsin (circle). Data represent mean ± s.e.m of three independent experiments. (B) Western blot analysis of A/PR/8/34 and A/Netherlands/602/09 HA cleavage after infection of A549 cells in presence or absence of plasminogen (PLG) or trypsin (Try). Membranes were probed with anti-HA and anti-tubulin antibodies. kDa (apparent molecular weight). NI stands for uninfected. (C) Infectious A/PR/8/34 (n = 3–5) and A/Netherlands/602/09 (n = 3) lung virus titers at the indicated time points post-inoculation of WT (black bars) or PLG-KO mice (white bars). Data represent mean ± s.e.m of 3–5 individual mice per group. n = mice per group and per time-point.

To gain further insight into the role of plasminogen in virus replication, A549 cells were infected with IAV in the absence or presence of plasminogen. Interestingly, plasminogen supported the replication of IAV A/PR/8/34 but not that of A/Netherlands/602/09 (Figure 2A). In contrast, trypsin supported replication of both viruses while no replication was observed in absence of proteases. Since plasminogen promotes IAV replication through HA cleavage [11], plasminogen-mediated HA cleavage of both viruses was compared (Figure 2B). In absence of proteases (−), HA0 precursor protein was detected in A549 cells infected with either virus. In presence of plasminogen (PLG), an additional band, corresponding to HA2 [11] was detected at 25 kDa in A/PR/8/34, but not in A/Netherlands/602/09 infected cells. In presence of trypsin (Try), HA2 was detected in cells infected with either virus. Similar levels of tubulin were detected, which was included as control cellular protein. Thus, plasminogen promotes cleavage of HA of IAV A/PR/8/34 but not that of A/Netherlands/602/09, which correlated with differences in replicative capacity of these viruses in presence of plasminogen.

On day 2 post-inoculation with IAV A/PR/8/34, mean lung virus titer of PLG-KO mice was significantly lower than that of WT mice (Figure 2C). This difference was not observed for IAV A/Netherlands/602/09. For both viruses, and at the other days post-infection, no significant differences in lung virus titers were observed between PLG-KO and WT mice. Thus, *in vivo*, plasminogen promoted early virus replication of IAV A/PR/8/34 but not of A/Netherlands/602/09. Since the absence of plasminogen protected mice against both viruses, the deleterious effect of plasminogen was most likely independent of virus replication in the lungs.

### Pulmonary injury and virus dissemination

**Figure 3:**
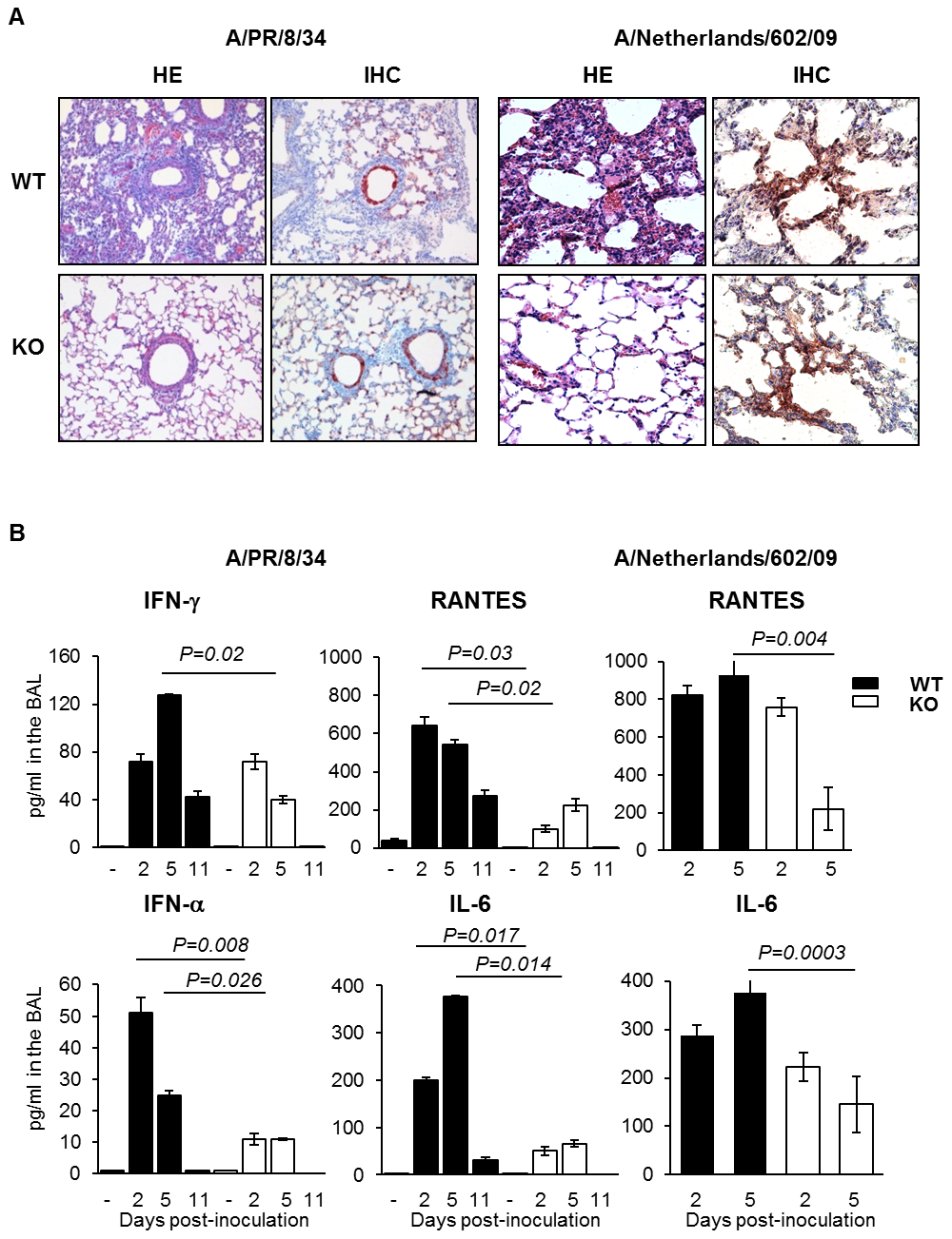
Plasminogen-deficiency prevents severe inflammation. (A) Histopathological analysis of lungs from infected WT and PLG-KO mice inoculated with A/PR/8/34 virus (day 3 post-infection) or A/Netherlands/602/09 virus (day 5 post-infection). Thin sections of lungs obtained from infected and uninfected WT and PLG-KO mice (as indicated) were stained with hematoxilin end eosin (HE) to evaluate histopathological changes. Note the marked infiltration of inflammatory cells in the lungs of infected WT mice, which was largely absent in the lungs of PLG-KO mice. The results shown are representative for two-three mice for both groups. Immunohistochemistry (IHC) using a monoclonal antibody for the influenza A virus nucleoprotein was used to detect virus-infected cells. Cells positive for the presence of viral antigen stained red. (B) Cytokine levels in BAL were assessed by ELISA on the indicated days post inoculation of WT (black bars) and PLG-KO mice (white bars) with IAV A/PR/8/34 or A/Netherlands/602/09. Data represent mean ± s.e.m. of 3–6 mice per group.

To assess possible other contributions of plasminogen to the pathogenesis of IAV infections, inflammation of the lungs and viral dissemination were examined after infection of mice with IAV A/PR/8/34 or A/Netherlands/602/09. At day 3 post-infection, extensive alveolar damage and marked cellular infiltrates were observed in lungs of WT mice in contrast to those of PLG-KO mice (HE) after A/PR/8/34 virus infection (Figure 3A, left panel). This difference was also observed upon infection with A/Netherlands/602/09 virus, at day 5 (Figure 3A, right panel) but not at day 3 post-inoculation (data not shown). For all conditions, in WT and PLG-KO mice, similar numbers of IAV-infected cells were detected by immunohistochemistry (IHC). Also, no lesions were observed in Mock-infected mice (data not shown). Thus, plasminogen-deficiency protected mice against inflammation induced by A/PR/8/34 and A/Netherlands/602/09 viruses, showing that plasminogen plays a deleterious role in lung inflammation, independent of virus replication in the lungs.

**Figure 4:**
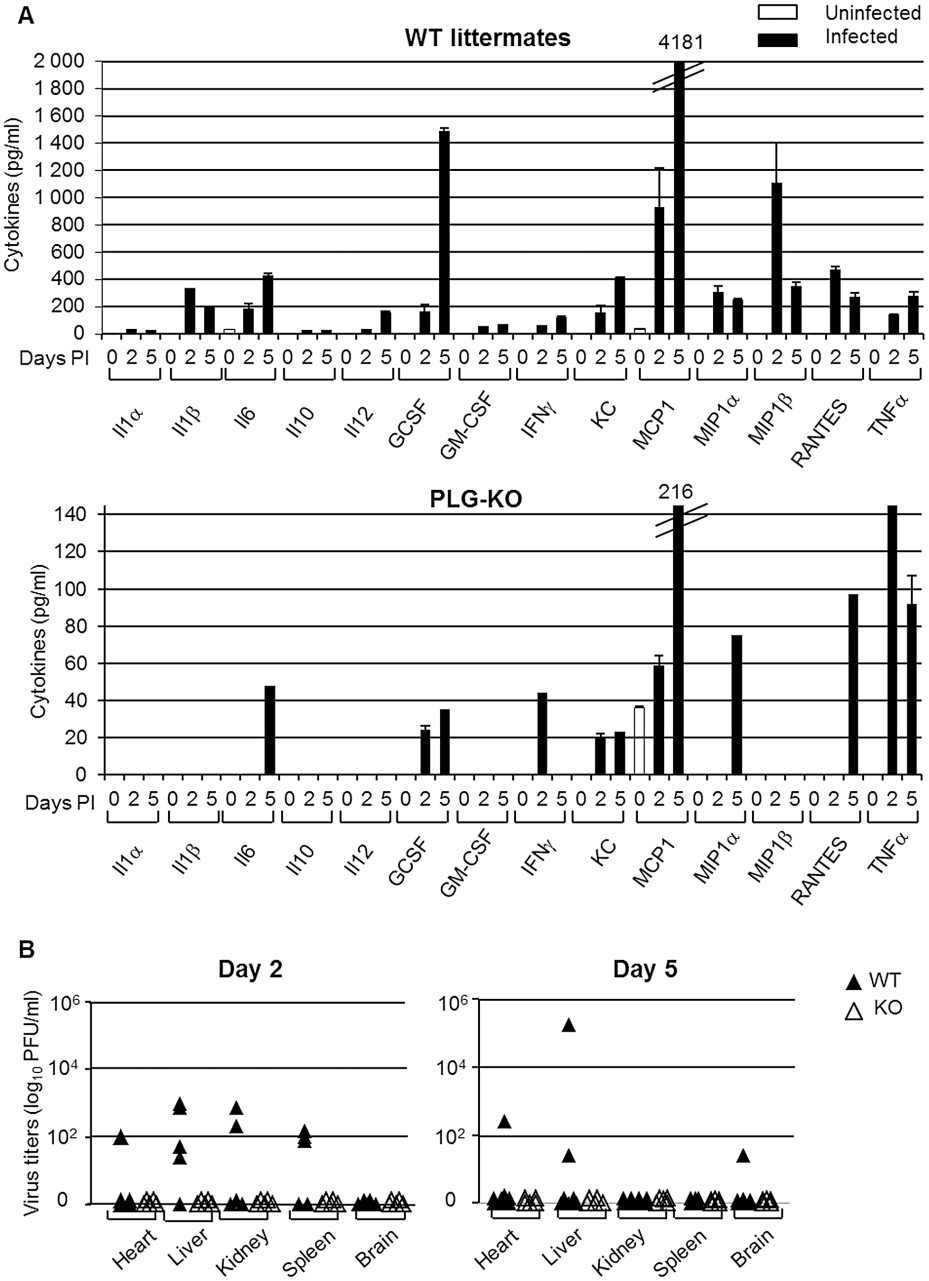
Plasminogen-deficiency prevents severe inflammation and virus dissemination. (A) Cytokine levels in BAL were assessed by 23-multiplex Luminex kit (uninfected, white bars; infected, black bars) on the indicated days post inoculation of WT (top panel) and PLG-KO mice (bottom panel) with IAV A/PR/8/34. The levels of IL-2, IL-3, IL-4, IL-5, IL-9, IL-12(p70), IL-13, IL-17 and eotaxin were below the detection limit (not shown). Data represent mean ± s.e.m. of 2 individual mice per group from one experiment and is representative of 2 individual experiments (total n = 3–6 mice per group). (B) A/PR/8/34 virus titers in the indicated organs of WT (closed symbols) and PLG-KO mice (open symbols) was assessed 2 and 5 days post-inoculation.

To investigate the difference in pulmonary inflammation between PLG-KO and WT mice, cytokine levels in the bronchoalveolar lavages (BALs) were assessed by ELISA (Figure 3B) or a luminex-based cytokine detection assays (Figure 4A) at various time point post-infection. Upon inoculation of A/PR/8/34 virus, both in PLG-KO and WT mice, BAL cytokine levels increased 2 and 5 days post-inoculation. However, in BAL of PLG-KO mice cytokine levels were considerably and significantly lower than in those of WT littermates (see scale differences for Figure 4A), which correlated with reduced IAV-induced lung inflammation in absence of plasminogen. Upon A/Netherlands/602/09 virus infection, release of cytokines in the BAL was also significantly higher in WT mice compared to PLG-KO mice at day 5 but not at day 2 post-inoculation (Figure 3B, right panel). Thus in concordance with the histological analysis, plasminogen promoted lung inflammation of IAV A/PR/8/34 and A/Netherlands/602/09 viruses, showing that the effect is most likely independent of virus replication in the lungs. Furthermore, in PLG-KO mice the virus failed to disseminate to extra pulmonary organs unlike in WT mice, upon intranasal infection with A/PR/8/34 virus (500 PFU) (Figure 4B). Especially high virus titers were detected in the liver, the source of plasminogen. Collectively, these results suggest that plasminogen plays an important role in promoting the inflammatory response and virus dissemination to extra-pulmonary organs during IAV-infection.

### Fibrinolysis and IAV pathogenesis

**Figure 5:**
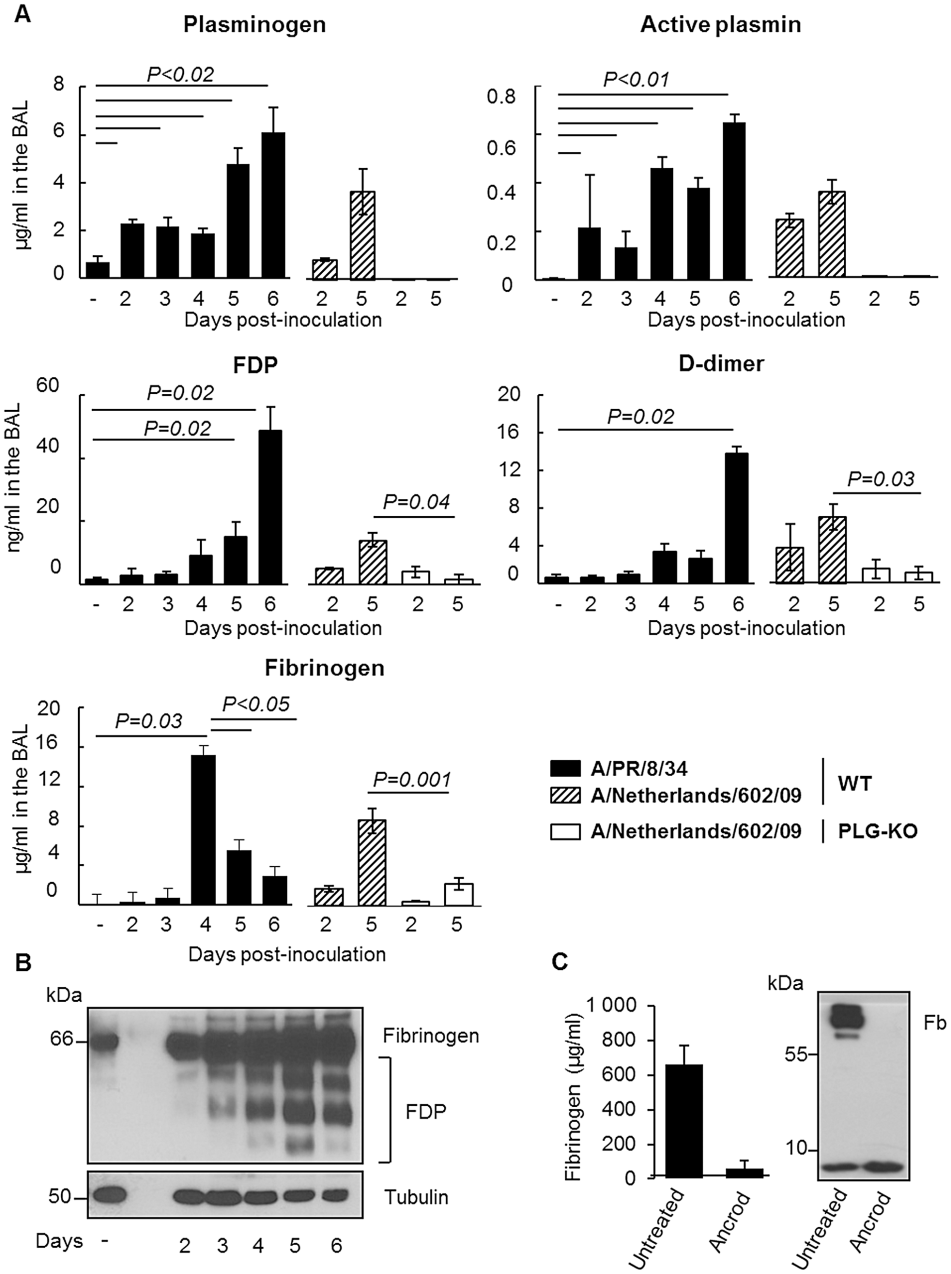
Fibrinolysis is induced following severe influenza infections. (A) Levels of Plasminogen, Active Plasmin, FDP, D-dimer and Fibrinogen, were determined by ELISA in the BAL of A/PR/8/34 infected or uninfected (−) C57BL/6 mice after the indicated days post-inoculation. Markers were also evaluated in the BAL of WT or PLG-KO mice infected with A/Netherlands/602/09 virus. Data represent mean ± s.e.m of n = 3–6 mice per group. (B) Western blot analysis for the detection of fibrinogen and FDP in the lungs of IAV-infected mice on the indicated days post inoculation (representative of n = 3). kDa: apparent molecular weight. n = mice per group. (C) Presence of fibrinogen was assessed in the blood of mice treated or not with Ancrod by ELISA (left panel) or Western blot analysis (right panel). The results represent the mean values ± s.e.m from 3 individual animals per group for the ELISA. The western blot analysis is representative for results of 3 mice per group.

Since degradation of fibrin is one of the main functions of plasminogen/plasmin, we hypothesized that the host fibrinolytic system plays a role in the pathogenesis of IAV infection. First, we investigated whether IAV infection induced fibrinolysis. To this end, mice were inoculated with IAV A/PR/8/34 and at various time points post-inoculation, the level of fibrinolysis markers in BALs was assessed by ELISA (Figure 5A). Plasminogen and active plasmin levels were barely detectable in the BAL of uninfected mice but their levels significantly increased during the course of infection. Levels of fibrinogen also significantly increased at day 4 post-infection and then dropped at days 5 and 6, suggesting a recruitment of fibrinogen to the lungs and a rapid consumption of the molecule and fibrinolysis. Finally, levels of FDP and D-dimers, degradation products of fibrinolysis, significantly increased upon infection, reaching 45 and 13 ng/ml respectively on day 6 post-inoculation. Similar results were also obtained upon infection with influenza virus A/Netherlands/602/09 (Figure 5A). As expected, in the BAL of infected PLG-KO mice, used as negative control, fibrinolysis markers were barely detectable. Thus, IAV infection induced fibrinolysis. These results were confirmed by Western blot analysis using an antibody directed against the mouse Aα chain of fibrinogen (Figure 5B), which recognizes purified mouse fibrinogen at a molecular weight of 66 kDa (data not shown). Compared to uninfected mice (−), fibrinogen was readily detectable 2–6 days post-inoculation in the lungs of infected mice. In the tissues, no marked fibrinogen consumption was detected but during the course of IAV infection, additional smaller bands corresponding to FDP were observed in mouse lungs. These findings confirmed that fibrinolysis took place during IAV infections *in vivo*.

**Figure 6:**
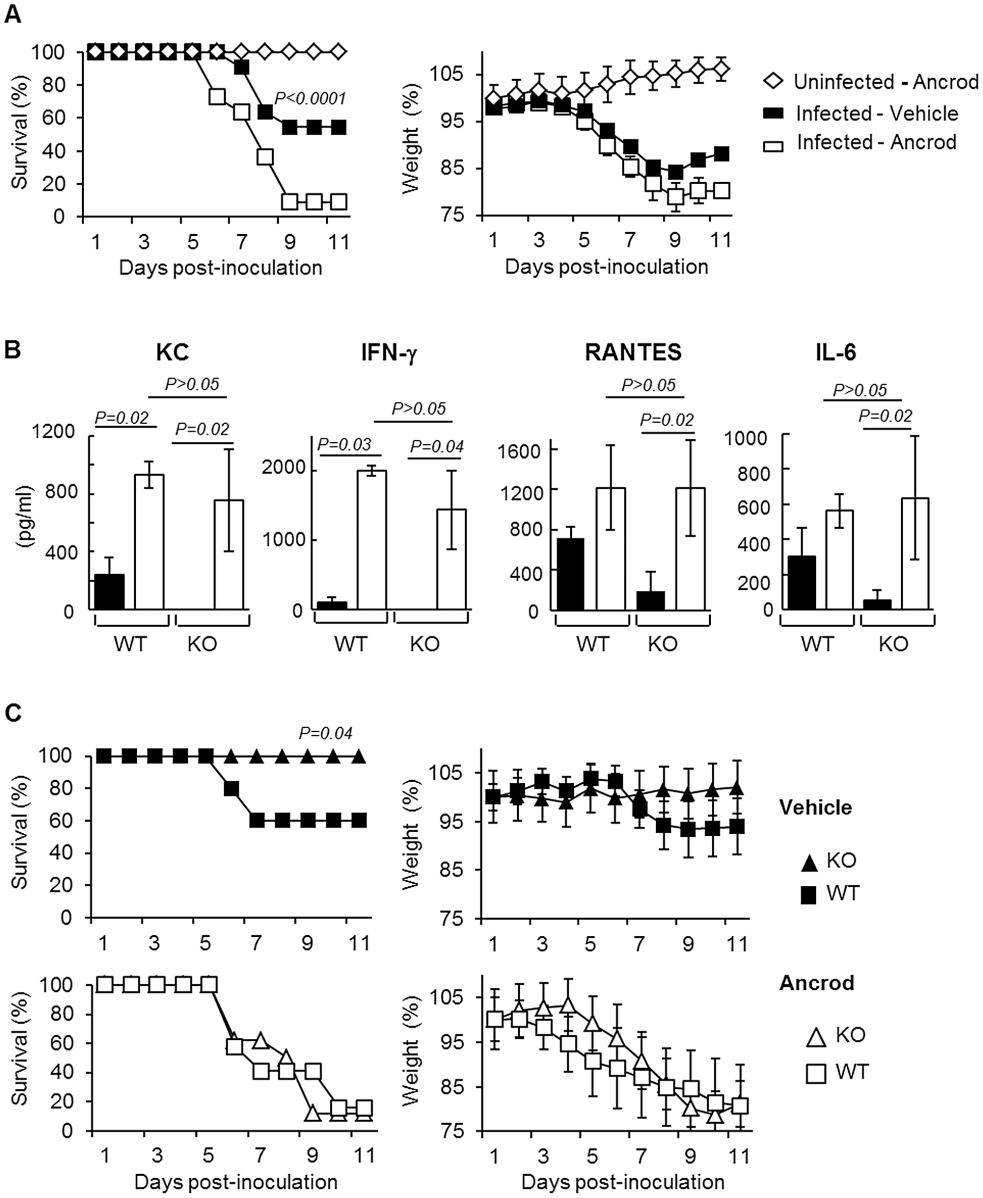
Effect of Ancrod treatment on inflammation and IAV pathogenesis. (A) Survival and weight loss of mice treated with Ancrod (open symbols, n = 11) or not (closed symbols, n = 11) after infection with IAV A/PR/8/34 (squares) or uninfected mice (diamonds, n = 5). Weight loss data represent weight average ± s.e.m of the above indicated number of mice. (B) Cytokines levels in the BAL were measured by ELISA after A/PR/8/34 infection of WT and PLG-KO (KO) mice treated with Ancrod (white bars) or not (black bars). Data represent mean ± s.e.m. of n = 4 mice per group. (C) Survival rate (left panels) and weight loss (right panels) of WT (squares) and PLG-KO (triangles) mice treated with Ancrod (open symbols) or not (closed symbols) after intranasal inoculation with IAV A/PR/8/34 (n = 8–10 mice per group). Weight loss data represent weight average ± s.e.m of the above indicated number of mice.

To simulate the depletion of fibrin (and therefore fibrinolysis), mice were treated with the snake venom Ancrod, a thrombin-like protease that cleaves the Aα chain of fibrinogen, enhancing its degradation and severely reducing its plasma levels (Figure 5C). Treatment with Ancrod significantly increased IAV-induced weight loss and mortality compared to vehicle-treated mice, but had no effect on uninfected control mice (Figure 6A). This increased mortality was also associated with an increase in inflammation of the lungs, as detected by elevated cytokine levels in the BAL (Figure 6B, WT). Of particular interest, the level of interferon-gamma was barely detectable in untreated mice but severely increased upon ancrod treatment. Thus, degradation of fibrin(ogen) contributed to inflammation and increased pathogenicity of IAV infection.

### Plasminogen promotes IAV pathogenesis through fibrinolysis

Next, we investigated whether Ancrod treatment could reverse the protective effect of plasminogen-deficiency in terms of inflammation and mortality rate. Again, PLG-KO mice were protected from lung inflammation (p<0,05, between WT versus PLG-KO), as judged from cytokine responses (Figure 6B) and from IAV-induced mortality (Figure 6C). Interestingly, Ancrod-treatment reversed the protection observed in the absence of plasminogen and cytokine responses and mortality rates were similar to those of Ancrod treated WT mice (Figure 6B and C, p>0.05, between WT-treated and PLG-KO-treated ancrod). Ancrod had no effect in uninfected mice (Figure S1). Thus, fibrinolysis contributes to inflammation and pathogenesis of IAV infections, which is mediated by plasminogen.

**Figure 7:**
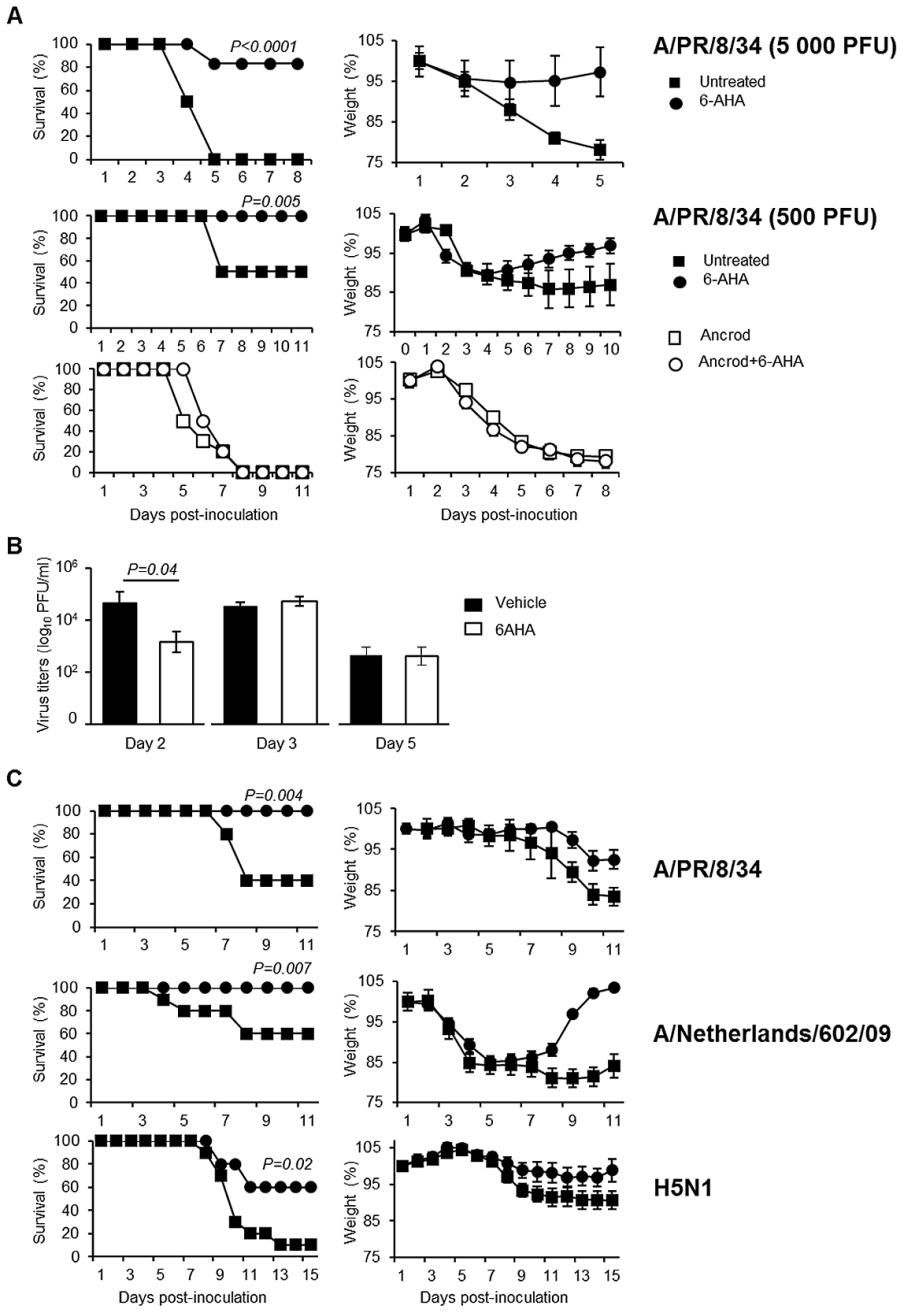
Effect of 6-aminohexanoic acid and/or Ancrod treatment on the course of IAV infection. Survival and weight loss of IAV inoculated C57BL/6 mice treated with 6-AHA (circle) or not (squares). (A) Mice were inoculated with IAV A/PR8/34 (5,000 PFU, n = 28 or 500 PFU; n = 11) in presence (open symbols) or absence (closed symbols) of Ancrod. 6-AHA treatment was initiated on the day of inoculation. (B) Infectious A/PR/8/34 lung virus titers in 6-AHA treated or untreated mice. Data represent mean ± s.e.m of 3 individual mice per group. (C) Mice were inoculated with IAV A/PR/8/34 (n = 10), A/Netherlands/602/09 (n = 16) or A/chicken/Ivory-Coast/1787/2006 (n = 10) as indicated. 6-AHA treatment was initiated two days post-inoculation. n = per group. Weight loss data represent weight average ± s.e.m of the above indicated number of mice.

To further confirm if the deleterious role of plasminogen is caused by fibrinolysis, we tested the outcome of infection of mice after treatment with Ancrod and/or 6-aminohexanoic acid (6-AHA). Indeed, 6-AHA is a lysine analogue that binds to the lysine binding sites of plasminogen, inhibiting plasminogen-binding to fibrin(ogen) and plasmin-mediated fibrinolysis [17]. First, 6-AHA treated mice inoculated with 5,000 or 500 PFU of A/PR/8/34 were significantly more resistant to infection than untreated mice (Figure 7A) and this protection correlated with reduced inflammation in 6-AHA treated animals (Figure S2). Also, lung virus titers were significantly lower in 6-AHA-treated mice compared to untreated mice, at day 2 but not at days 3 or 5 post-infection (Figure 7B). Thus, inhibition of plasminogen fibrinolytic activity protected mice from developing pneumonitis and severe disease. Furthermore, Ancrod-treatment of 6-AHA treated mice over-rode the protective effect of 6-AHA, again resulting in IAV-induced mortality (Figure 7A, lower panel). Administration of Ancrod and/or 6-AHA had no effect in uninfected mice (Figure S3). Thus, the protective effect of 6-AHA was reversed by Ancrod-mediated fibrinogen degradation, demonstrating that plasminogen contributed to pathogenesis of IAV infection through fibrinolysis activation.

### 6-AHA protects against influenza

Preventing deleterious inflammation after IAV infection could be a promising new strategy to treat IAV infections. Therefore, we investigated whether blocking the fibrinbolytic activity of plasminogen by 6-AHA administration at a later time point post-inoculation was still protective. WT mice were inoculated with IAV A/PR/8/34 and treated or not with 6-AHA, two days later. As shown in Figure 7C, treatment with 6-AHA improved the outcome of infection and prevented mortality. 6-AHA treatment also protected mice from infection with A/Netherlands/602/09 and highly pathogenic H5N1 viruses (Figure 7C, lower panels). Thus, blocking plasminogen-mediated fibrinolysis protected mice against infections with various and highly pathogenic IAVs.

## Discussion

The present study showed for the first time that fibrinolysis plays a central role in the inflammatory response and the pathogenesis of IAV infections. Consistently, evidence is accumulating that the fibrinolytic molecule plasminogen and plasmin are critical host factors for immune cell infiltration and cytokine production upon injury [18]–[20]. The absence of plasminogen blunts inflammation in response to several inflammatory stimuli and suppresses development of lesions [21]–[23]. In our study, absence of plasminogen also considerably reduced the extent of lung inflammation upon IAV infection. Since severe inflammation contributes to the pathogenicity of IAV infections of humans [2], [4], most likely the proinflammatory properties of plasminogen play a role in the pathogenesis of these infections. IAV have the capacity to bind plasminogen and convert it into its active form plasmin through viral or cellular proteins like annexin-2 [11], [12]. However, the extent of plasminogen activation is strain-dependent [11], which may explain differences in pathogenicity of IAV strains.

Mechanistically, the mode of action of plasminogen-driven lung inflammation was through fibrinolysis. Indeed, degradation of fibrinogen by Ancrod treatment increased pathogenicity of IAV infection and compensated the protective effect in PLG-KO mice or in mice in which plasminogen fibrinolytic activity was blocked by 6-AHA treatment. Consistently, Keller et al showed an activation of the fibrinolytic system during non-pathogenic IAV infection in mice [15]. Remarkably, in humans increased production of D-dimer, a marker of fibrinolysis was found to be a risk factor for fatal outcome of H5N1 and pandemic H1N1 virus infections [24], [25]. Furthermore, IAV infections have been associated with bleeding medical disorders [26], [27]. Thus, as for bacteria [28], the dysregulation of hemostasis by virus infections may cause serious complications. Consistent with our results, it was recently demonstrated that endothelial cells are central orchestrators of cytokine amplification during IAV infections [29]. Interestingly, plasminogen-dependent inflammation appears early after infection with influenza virus A/PR/8/34, of which virus replication is promoted by plasminogen. In contrast, replication of influenza virus A/Netherlands/602/09 is independent of plasminogen and control of plasminogen activity has a delayed impact on inflammation and disease. Thus, the capability of plasminogen to cleave HA and promote virus replication may also contribute to lung inflammation for some IAV strains. Possibly, a sustained high degree of inflammation is deleterious for the host.

**Figure 8:**
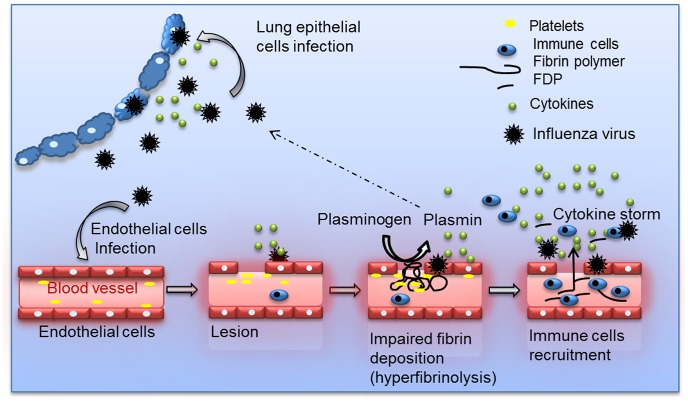
Schematic overview of the proposed model for Plasminogen-mediated influenza virus pathogenesis. During IAV infection, plasminogen is converted into plasmin. On the one hand, plasmin cleaves and activates the viral hemagglutinin, promoting IAV replication for some influenza strains. On the other hand, plasmin promotes inflammation via fibrinolysis and increases permeability.

Collectively, we propose a model (Figure 8) in which plasminogen-mediated fibrinolysis increases FDP production and vascular permeability allowing increase recruitment of inflammatory cells at the site of infection. As a positive feedback loop, plasminogen mediated virus replication may also further contribute to lung inflammation. Fibrinolysis may also allow systemic haematogenous spread of virus. Consistently, we and others detected IAV replication in extrapulmonary organs in plasminogen-competent mice [30]. Since plasminogen is omnipresent in the blood, it may provide certain IAV an alternative mechanism of HA cleavage in extra-pulmonary organs [10], [11]. For example, the plasminogen-binding property of the neuraminidase of A/WSN/33 strain is a determinant of its neurotropism and pathogenicity in mice [12], [13]. Interestingly, particular high virus titers were found in the liver, which is the primary source of plasminogen. This may explain why IAV can replicate in hepatocarcinoma liver HEPG-2 cells in the absence of exogenous proteases (Figure S4). Whether plasminogen-dependent IAV replication contributes to damage of the liver or other extra-pulmonary organs, as observed in Reye's syndrome or other postinfluenza complications [31] requires further investigation. Interestingly, differences in virus replication were not at the basis of plasminogen-dependent differences in pathogenesis of IAV infection although it also can contribute to exacerbation of inflammation. Indeed, A/Netherland/602/09 virus replication in the lungs was not affected by plasminogen deficiency, while infected PLG-KO mice were protected from infection. This is consistent with a recent report showing that presence of critical residues in HA, necessary for cleavage by plasmin is strain-dependent [32]. In addition, the HA of A/chicken/Ivory-Coast/1787/2006 contains a polybasic site, which is cleaved by furin-type proteases. This suggests that plasminogen plays a minor role in replication of this virus, while plasminogen deficiency still protected from infection with this virus. Alternative proteases may thus play a more dominant role in HA cleavage and virus replication *in vivo* than plasminogen [33]–[36].

For the clinical management of influenza patients, a limited number of antiviral drugs are available. The use of these currently available drugs is compromised by the emergence of virus strains that developed resistance to these drugs. Therefore, intervention strategies that aim at preventing deleterious inflammatory responses after IAV infection are of interest and do not suffer from resistance to antiviral drugs. Specifically, blocking protease activity may be an efficient way to achieve this, as previously suggested [37]–[39]. Our results are consistent with these studies but differ in term of mechanism of action. Indeed, our results suggest a more predominant role for proteases in lung hemostasis compared to virus replication and HA cleavage.

In summary, our findings reveal a previously unrecognized role for fibrinolysis and plasminogen in the pathogenesis of IAV infections. Thus, targeting plasminogen, its conversion into plasmin or regulating fibrinolysis may be a venue for the development of novel intervention strategies for the treatment of severe IAV infections.

## Materials and Methods

### Ethics statement

Experiments were performed according to recommendations of the “National Commission of Animal Experiment (CNEA)” and the “National Committee on the Ethic Reflexion of Animal Experiments (CNREEA)”. The protocol was approved by the committee of animal experiments of the University Claude Bernard Lyon I (Permit number: BH2008-13). All animal experiments were also carried out under the authority of license issued by “la direction des services Vétérinaires” (accreditation number 78–114). All efforts were made to minimize suffering.

### Reagent

Viruses, cells, and reagents used, were: IAV A/Netherlands/602/09 [40], A/chicken/Ivory-Coast/1787/2006 [41], A/PR/8/34 (American Type Culture Collection, ATCC), A549 cells (ATCC), Madin-Darby Canine Kidney cells (MDCK, ATCC), trypsin (Becton Dickinson), plasminogen and 6-AHA (Sigma), Ancrod (NIBSC), 23-Plex Mouse Cytokine Assay (Bio-Rad), ELISA kits for mouse -IL-6, -KC, -–RANTES, -IFN-α -IFN-γ(R&D Systems), -plasminogen (Mybiosource), -active plasmin (Kordia), -D-dimer, -fibrinogen and -FDP (Genway), antibodies anti-HA (Santa Cruz), anti-tubulin (Sigma), anti-NP (ATCC), anti-fibrinogen (Genway).

### In vitro experiments and proteins detection

Blood fibrinogen and lung proteins were extracted as described [42], [43] and proteins were analyzed by western blot [44]. A549 experiments were performed as described previously [11].

### Mice

Mice with a disrupted PLG gene (PLG-KO) and their WT littermates were bred as described previously [45]. Briefly, PLG heterozygous mice (C57BL/6 and 25% 129Sv) were crossed and WT and PLG-KO mice offspring were genotyped by polymerase chain reaction, which was performed, as previously described [46] using primers amplifying the WT PLG gene (5′ACTGCTGCCCACTGTTTGGAG 3′ and 5′ GATAACCTTGTAGAATTCAGGTC3′) or the inactivated PLG gene (5′ATGAACTGCAGGACGAGGCAG3′ and 5′ GCGAACAGTTCGGCTGGCGC 3′). Most of the experiments were performed using 5–6 weeks old mice. Also, males and females were used in the experiments. Groups between WT and PLG KO mice were homogenized for these different parameters. Except when PLG-WT and PLG-KO mice were used, experiments were performed with six-week-old C57BL/6 female mice (Charles River Laboratories).

### Mice infection and treatment

Mice were anesthetized with ketamine (42,5 mg/kg) and inoculated by the intranasal route with the indicated IAV in a volume of 25 µl. Upon inoculation, survival rates and loss of body weight was scored daily, as previously described [47]. For weight loss curves, the last measured value was carried forward until the end of the observation period. Alternatively, mice were sacrificed at various pre-fixed time points post-inoculation to perform bronchoalveolar lavages (BAL) or to sample organs. Virus titers in organs were determined by classical plaque assay using MDCK cells [47]. ELISA and luminex assays were performed according to the instructions of the manufacturer and virus titers were assessed as described [48]. Lungs histology and immunohistochemistry were performed as described [49]. Treatment with 6-AHA was injected intraperitoneally (30 mg per mouse in 200 µl of physiological serum) every 6 hours for 4 days. Ancrod was injected (1.75 unit per mouse) intraperitoneally two days before infection for 7 days at 10 hours intervals.

### Statistical analysis

Kaplan-Meier test was used for statistical analysis of survival rates and Mann–Whitney's test was used for lung virus titers and ELISA results, *p* values<0.05, were considered statistically significant. Two-tails analysis was performed. The number (n) of animals per experimental group is mentioned in the figure legends. Experiments were stratified in terms of weight, gender and age of the mice.

## Supporting Information

Figure S1 **Effect of Ancrod treatment on uninfected mice.** Survival and weight loss of uninfected PLG-KO mice treated with Ancrod (open triangle, n = 3).
(TIF)

Figure S2 **Effect of 6-AHA on cytokine levels in the BAL.** Cytokine levels in the BAL of IAV-infected C57BL/6 mice, treated or not (upper panel) with 6-AHA (lower panel) was evaluated by multiplex assay four days post-inoculation. Only detectable levels are shown. n = 3 mice per group. Please note the difference in scale of y-axis between treated and untreated animals.
(TIF)

Figure S3 **Effect of Ancrod treatment and/or 6-AHA treatment on uninfected mice.** Survival and weight loss of uninfected C57BL/6 mice treated with Ancrod and 6-AHA (open circles, n = 5) or 6-AHA only (closed circles, n = 5).
(TIF)

Figure S4 **IAV replication kinetics in HEPG-2 cells.** Replication kinetics of IAV A/PR/8/34 and A/Udorn/72 in absence of proteases was assessed after inoculating HEPG-2 cells at a MOI of 0.001.
(TIF)
