# Supplementary figures and images for: Plasminogen Controls Inflammation and Pathogenesis of Influenza Virus Infections via Fibrinolysis
Source: PLoS Pathog. 2013 Mar 21;9(3):e1003229. doi: 10.1371/journal.ppat.1003229 (PMC3605290; doi:10.1371/journal.ppat.1003229)

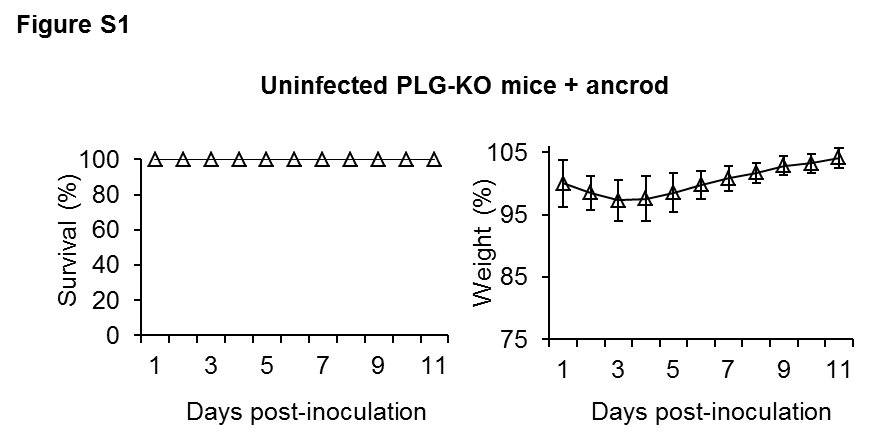

Supplement: Figure S1 — Effect of Ancrod treatment on uninfected mice. Survival and weight loss of uninfected PLG-KO mice treated with Ancrod (open triangle, n = 3). (TIF) [file ppat.1003229.s001.tif]

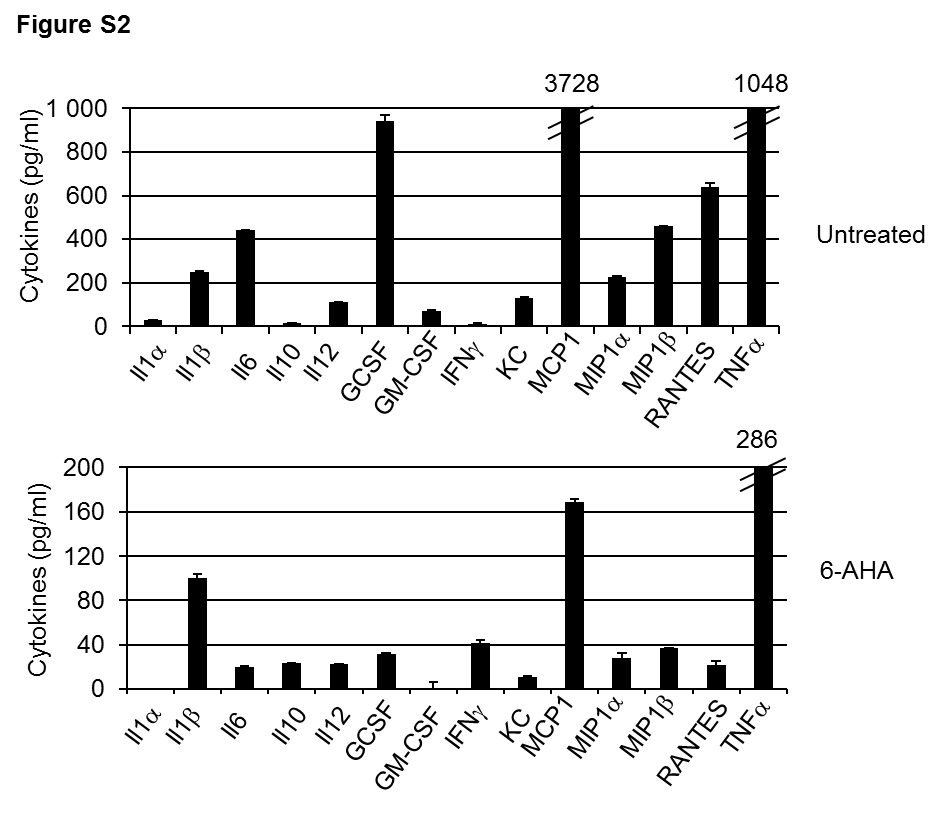

Supplement: Figure S2 — Effect of 6-AHA on cytokine levels in the BAL. Cytokine levels in the BAL of IAV-infected C57BL/6 mice, treated or not (upper panel) with 6-AHA (lower panel) was evaluated by multiplex assay four days post-inoculation. Only detectable levels are shown. n = 3 mice per group. Please note the difference in scale of y-axis between treated and untreated animals. (TIF) [file ppat.1003229.s002.tif]

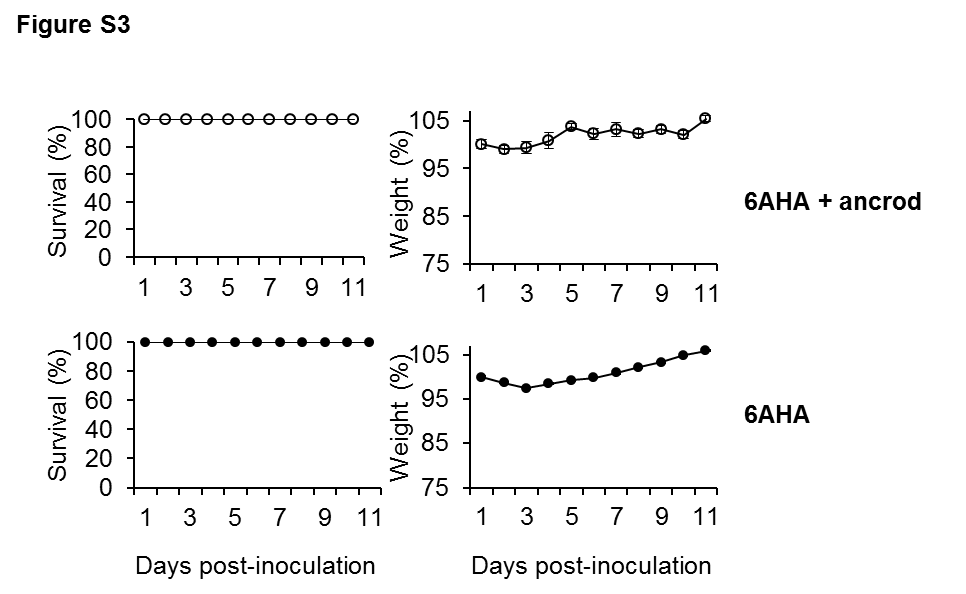

Supplement: Figure S3 — Effect of Ancrod treatment and/or 6-AHA treatment on uninfected mice. Survival and weight loss of uninfected C57BL/6 mice treated with Ancrod and 6-AHA (open circles, n = 5) or 6-AHA only (closed circles, n = 5). (TIF) [file ppat.1003229.s003.tif]

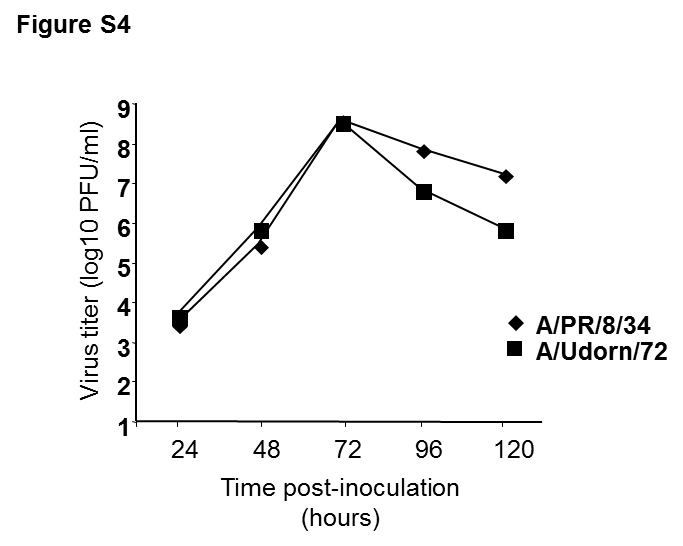

Supplement: Figure S4 — IAV replication kinetics in HEPG-2 cells. Replication kinetics of IAV A/PR/8/34 and A/Udorn/72 in absence of proteases was assessed after inoculating HEPG-2 cells at a MOI of 0.001. (TIF) [file ppat.1003229.s004.tif]
